# Putting Access to Veterinary Care on the Map: A Veterinary Care Accessibility Index

**DOI:** 10.3389/fvets.2022.857644

**Published:** 2022-04-04

**Authors:** Sue M. Neal, Mike J. Greenberg

**Affiliations:** ^1^Veterinary Care Accessibility Project, Rochester, MI, United States; ^2^Center for GIS and Spatial Analytics, West Chester University, West Chester, PA, United States

**Keywords:** access to care, veterinary geographic distribution, veterinary shortage, veterinary care index, veterinary staffing, veterinary care desert

## Abstract

Access to veterinary care is a complex problem that sits at the intersection of a number of societal factors including income inequality, access to transportation, language and cultural differences as well as the spatial distribution of veterinary care providers. This research aims to create an index evaluating accessibility of veterinary care across the contiguous United States and thus fill an important gap in the literature. The location and number of employees of veterinary clinics were aggregated at the county level. Projected pet population and household counts were used to normalize the number of employees to provide a relative assessment of the distribution of care access. Existing US Census data was used as percentile rankings to identify counties which may experience additional, non-spatial, barriers to care. By combining the percentile rankings of each of the variables, an overall index was created, evaluating the relative accessibility of veterinary care in each of the counties of the contiguous US. This work can be used by organizations looking to improve access to care or by policymakers considering legislation that impacts this issue. It may also be of use to individuals in human health care as they consider the intersection of human wellness and companion animal wellness.

## Introduction

Over time, there has been a steady increase in the number of companion animals that are not receiving veterinary care with cats being the most affected (from 32 to 45% in the period between 1998 and 2011) (1). During this same period, the cost of veterinary medical care has been rising faster than wider inflation but also faster than the rapidly increasing costs of human health care (2). The average American spends 47% more on equivalent veterinary care today than a decade ago (3). The functional impact is that a lower proportion of people are seeking care for their pets (4) resulting in what is considered the greatest current threat to companion animal welfare in the US (5).

Cost of veterinary care is an often cited barrier to veterinary care (3, 6). It was also cited as the most common reason individuals chose to use nonprofit spay and neuter clinics over private practice clinics (7). A major study by Bayer in 2011 highlighted an alarming decrease in the demand for veterinary care (6) with a follow up study completed in 2014 which showed increasing client citing of cost and stress to animals as reasons for not visiting veterinarians, especially for preventative care (8). A full review of the articles published over nearly two decades indicated that fully 61% of articles published on access to care issues included cost as part of their article (9). Challenges of discussing financial issues with pet caregivers is also cited as a primary source of job-related stress for veterinarians (10). What results is a conflict for veterinarians who have to consider the suffering and impacts to an animal's quality of life and a client's ability to pay for needed care (10).

The issue of cost as a barrier is also not limited to low income individuals. Researchers have found economic barriers to care existing at poverty, low income and mid income levels (5) and across racial and ethnic groups (11, 12). The AVCC (5) report identifies the most common barriers to veterinary care as self-reported through a survey. Distance to the vet clinic, veterinary care cost and transportation in general all emerged as significant barriers in this research (5). Additionally, barriers to affordability of care are reported in urban regions as well as remote, rural regions where care centers are sometimes not available at all (13). Lack of veterinary facilities, or an inability for individuals to physically access facilities is cited as an additional barrier to care (3).

Cultural differences or a lack of awareness of the need for companion animals to receive veterinary care is another possible barrier that might co-occur with economic barriers in low-income communities (7, 14). Language barriers are also commonly cited as a barrier in provision of care in human health (15) and it is likely that similar challenges exist in other care settings, such as veterinary care. In a recent survey, only 8% of veterinary clinics reported having staff that could speak Spanish fluently and that language challenges decreased satisfaction with veterinary experiences (16). Considering income/poverty status alone in determining who faces access to veterinary care (hereafter A2C) barriers thus is an inadequate measure of needs assessment (7).

When caregivers are unable to access veterinary care for their companion animals, they may be forced to rehome/surrender them to a shelter (17), euthanize them (18), or avoid obtaining a pet in the first place and thus lose out on the benefits of having a companion animal (19). For example, Weiss et al. (17) found that upwards of 40% of individuals rehoming animals indicated that access to free or low cost veterinary care would be something that would've helped them retain their pet.

There is an identified lack of geographic research that explores access to veterinary care issues (9). Geographic Information Systems is a powerful tool to visualize the distribution of veterinary facilities relative to the socio-economic status and other barriers to care. This research aims to fill the existing gap in the literature through the creation of a spatial index that incorporates the main variables impacting access to veterinary care.

## Materials and Methods

The objective of this research is to introduce a spatial index that assesses the relative risk of experiencing barriers to accessing veterinary care for companion animals across the contiguous United States at the county level. An aggregate percentile rank variable was derived for each county using the data and methods described below. Variables for entry in the index were chosen based on the existing research around barriers in access to veterinary care as discussed in the introduction.

### Scope and Scale

The geographic unit of analysis is at the county level. Counties are a familiar unit of analysis for communicating data to the public. Access to Care for human populations is often measured and reported at the county level in this context providing a comparative justification for this unit. The Robert Woods Johnson County Health Rankings is one example (20). As some organizations move toward a One Health model that includes companion animal wellness as part of the human wellness continuum of care, using a similar geographic unit of analysis will add value in how the results of this analysis can be used as part of existing human based A2C maps and datasets. One Health acknowledges the link between non-human animal wellness and human wellness including the psychosocial value of the human-animal bond, zoonotic disease transmission and other factors (21). The proposed index covers the contiguous United States because the pet demographic data used in the analysis did not include values for Alaska and Hawaii.

### Data

#### Socioeconomic Variables

The Centers for Disease Control (CDC hereafter) originated the Social Vulnerability Index (SVI hereafter) as a tool for evaluating the relative vulnerability of populations across the United States during times of disaster (22). The index contains a number of different variables organized under four main themes. Variables, expressed as percentile ranks, were chosen for this research using the 2018 version of the SVI. The variables were chosen based on their relevance to barriers to veterinary care identified in the extant literature as discussed in the introduction. The rank percentiles of the number of people in poverty, the per capita income, the number of people with no access to a vehicle and the number of people who speak English less than well were selected to enter into our analysis. The 2018 SVI was accessed through Living Atlas in ArcGIS Online at the county level. The SVI data is built using Census derived data, see Flanagan et al. (22) for a detailed discussion of the variables and the methods used to calculate them (22).

#### Veterinary Coverage Variable

The coverage of veterinary care is conceptualized as the aggregate number of veterinary clinic employees normalized by the predicted pet population in any given county in the US and expressed as the number of clinic employees per 1,000 pets. The number of employees is used in lieu of the number of clinics due to the range of sizes of veterinary clinics that would impact their functional capacity to provide coverage for any given population. This includes all type of employees, ranging from administrative support staff to veterinary assistants and technicians. While not all clinic staff are engaged in delivery of direct care, additional supporting staff may increase the capacity for care through efficiency gains [see for example (23)]. Clinics can range in size from small, single veterinarians with limited support staff to large corporate-owned facilities with several veterinarians and numerous support staff (24). This composite variable was created using the data described in the following two subsections.

#### Veterinary Employees

Veterinary clinic locations and number of employees were obtained using ESRI's GIS online suite of applications. Veterinary clinics were defined using the North American Industry Classification System. The North American Industry Classification System (hereafter NAICS) provides a standardized method for classifying industries across the continent of North America (25). For purposes of this research, the NAICS code 541940 was used which captures all types of veterinary clinics. According to the NAICS definition: “This industry comprises establishments of licensed veterinary practitioners primarily engaged in the practice of veterinary medicine, dentistry, or surgery for animals; and establishments primarily engaged in providing testing services for licensed veterinary practitioners (26)”. While they are technically part of the 541940 code, businesses listed as laboratory testing services facilities were removed from the results since they do not provide direct services to companion animals and may serve a large geographic area.

The database that the business info drawn from is maintained by ESRI through data gathered by Infogroup (27). Infogroup sources authoritative business data on a large number of industries in the US which are then geocoded for mapping purposes (27). The vintage of the data accessed are January of 2020 for the clinic employee counts and April of 2020 for the clinic locations (28).

#### Pet Population

For purposes of this research, “pets” are defined as household cats and dogs. Estimating the population of companion animals at the county level can be challenging. There is not one single approach to doing this that is standardized and broadly agreed upon (29). A number of pet demographic surveys have been completed, most notably the routine surveys conducted by the American Veterinary Medical Association (AVMA hereafter) and the American Pet Products Association. Other smaller scale social science surveys have been completed [see Applebaum (29), for a detailed comparison and discussion]. Using the General Social Survey as one additional approach that was recently advanced (29).

For purposes of this research, the 2017–2018 AVMA Pet Demographic survey was used because it is generally recognized within the veterinary industry, accessible through the AVMA and periodically repeated to update the data. The AVMA survey reports, among other things, estimates of pet ownership rates and total estimated population of cats and dogs at the state level. State total pet populations were directly extrapolated for use at the county level in this research. While this is imperfect it provides a first step toward understanding the spatial variability in the proposed index. See the AVMA Pet Demographic Sourcebook for a detailed discussion of their methodology (executive summary publicly available at: AVMA-Pet-Demographics-Executive-Summary.pdf with full report available through the AVMA).

The household count estimate from 2016 (to match the vintage of the AVMA survey) at the county level was obtained through ArcGIS online [see ESRI documentation for explanation of methodology and data sources (30)]. The 2019 household count was also obtained from the same source.

### Methods

#### Ratio of Veterinary Employees to Pet Population

The AVMA total pet population estimate for each state was divided by the state's Census estimate 2016 household count and then multiplied by the 2019 household count at the county level. This was used to represent the projected pet population at the county level in order to have a method to normalize the number of veterinary employees. Normalized Intensive Statistics provide a way to present data in comparative form by dividing the raw value by a given basis, a common tool in mapping (31). Further, normalization is recommended when the resulting visualization is symbolized as a choropleth map (31).

#### Ranking Scores

Rank percentiles were calculated for each of the variables entered into the index. Rank percentile is defined as the proportion of scores in a distribution that an individual score is greater than or equal to. Already calculated values for the variables obtained from the SVI were used. For the veterinary employee to pet population ratio, the values were ranked in order from lowest to highest since lower levels of employees are equated with higher vulnerability to keep ranking logic consistent with the Social Vulnerability Index. For county level aggregated index ratings, the scores were ranked in order from highest to lowest. Percentile ranks were calculated with the following equation:


$$\begin{array}{l} Percentile\;Rank=\frac{Rank-1}{N-1} \end{array}$$

where *N* = the total number of data points. For the veterinary employee to pet population variable, all sequences of ties are assigned the average of the corresponding ranks so as not to underweight the more frequent zero value. For the county level percentile ranks the smallest of the corresponding ranks was used for any sequences of ties.

Once percentile rank calculations were completed for the veterinary coverage all of the percentile ranking values were summed at the county level. Then a composite percentile ranking was calculated for a final index value that was visualized in ArcGIS online. As such, the resulting index values are relative and not absolute. This approach is used because of the lack of research around the level of veterinary employee coverage that is optimal and the complex relationship between income and absolute affordability of care. In the resulting index, values approaching one have the highest access to veterinary care while those approaching zero have the lowest access to veterinary care. For ease in communicating the data, the fractional output was then multiplied by a factor of 100 such that 100 is the most accessible ranking and 0 the least. The resulting index represents the Veterinary Care Accessibility Score (VCAS, hereafter). Lastly, a state overview was created by compositing the values of all of the VCAS within each state and calculating a simple average.

## Results

### Veterinary Care Accessibility Score

**Figure 1:**
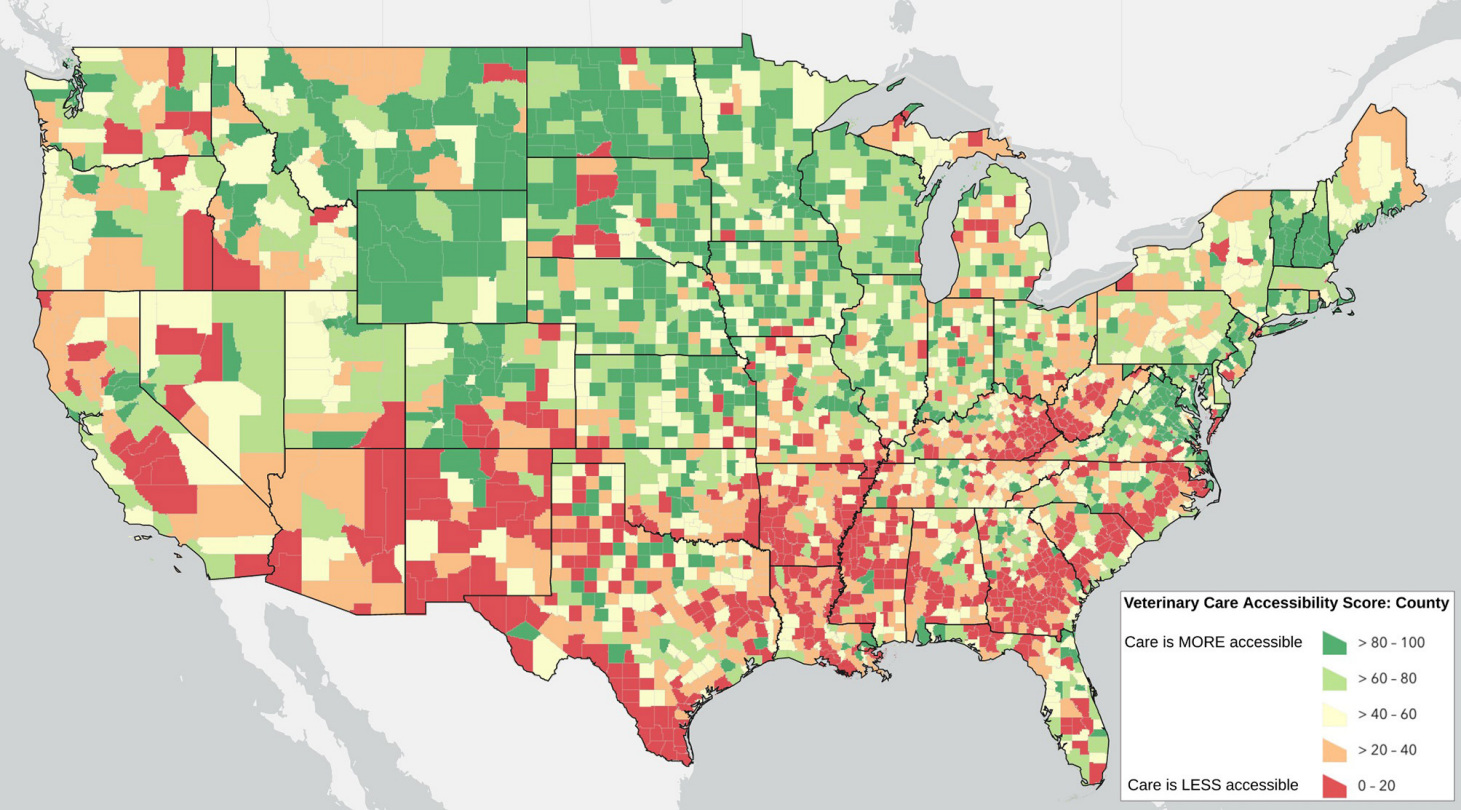
The Veterinary Care Accessibility Score: a relative measure of accessibility to veterinary care across the contiguous US. An online interactive and routinely updated map is available at: www.accesstovetcare.org

As discussed, the Veterinary Care Accessibility Score combines the percentile rankings for five factors that have been identified in the extant literature as impacting access to veterinary care. The resulting VCAS are displayed in Figure 1 as a choropleth map at the county level as quintiles.

**Figure 2:**
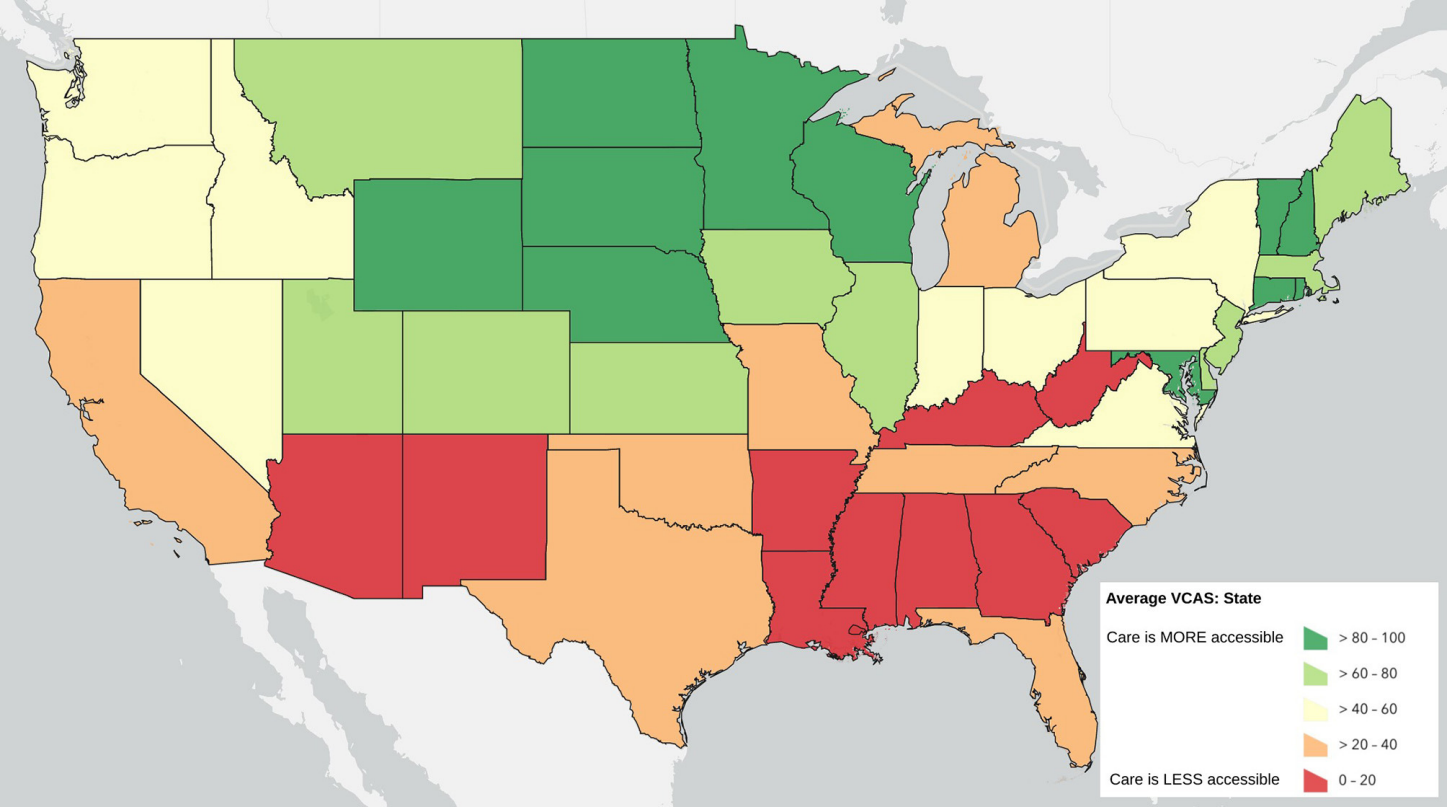
State Average Veterinary Care Accessibility Score by quintile.

Counties do not operate in isolation, however, and so there may be an added effect when there are multiple counties in a region that have lower levels of accessible veterinary care. Figure 2 shows the results of the state average VCAS symbolized by quintiles.

**Figure 3:**
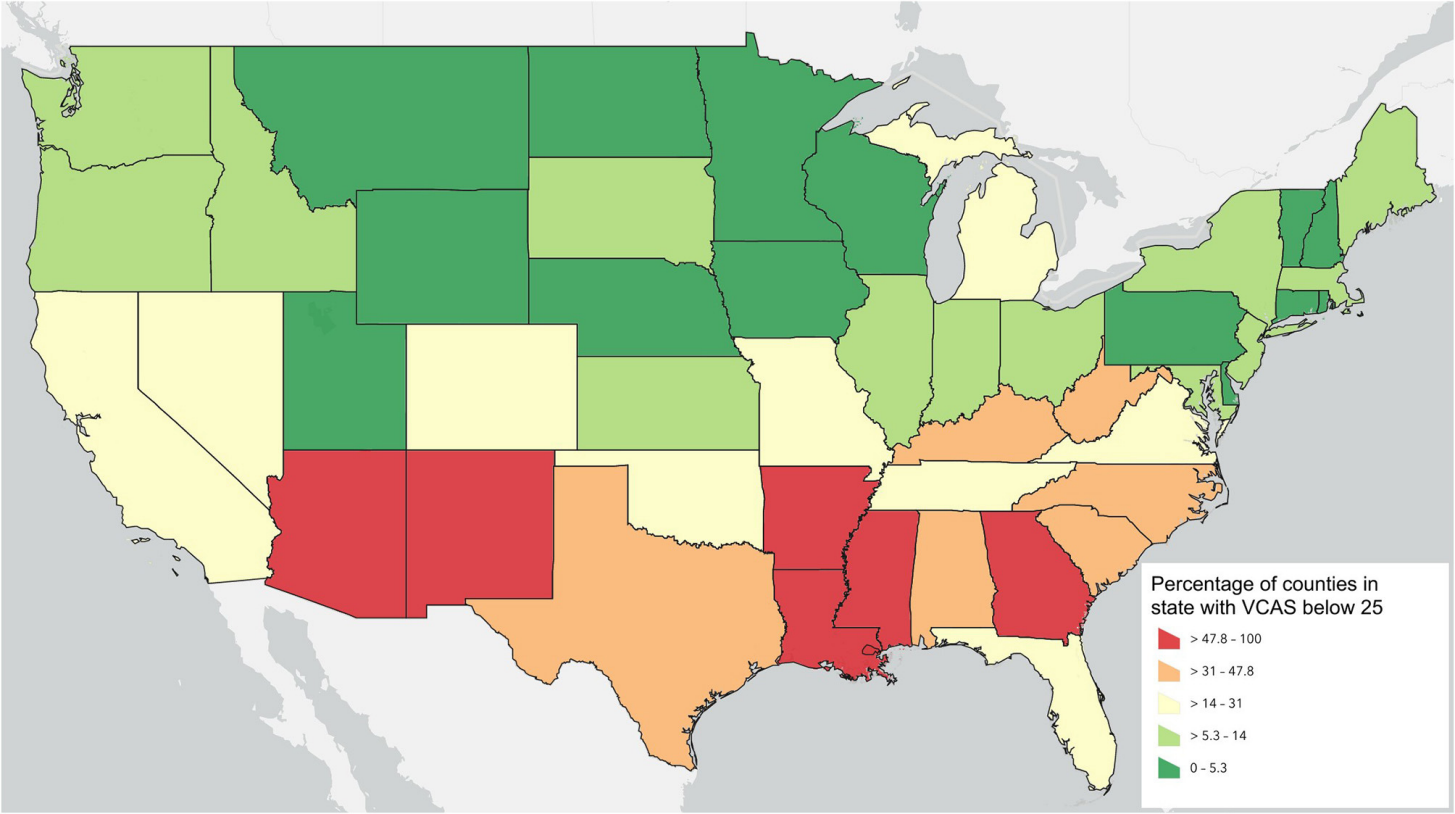
Proportion of counties falling in the lowest quartile of the national VACS by symbolized by quintiles.

Similarly, the proportion of counties that fall within the lowest quantile of the VACS can also given insight in to the challenges confronted at the state level. Figure 3 displays the proportion of counties in each state that fall at the lowest quartile of the national VCAS.

**Figure 4:**
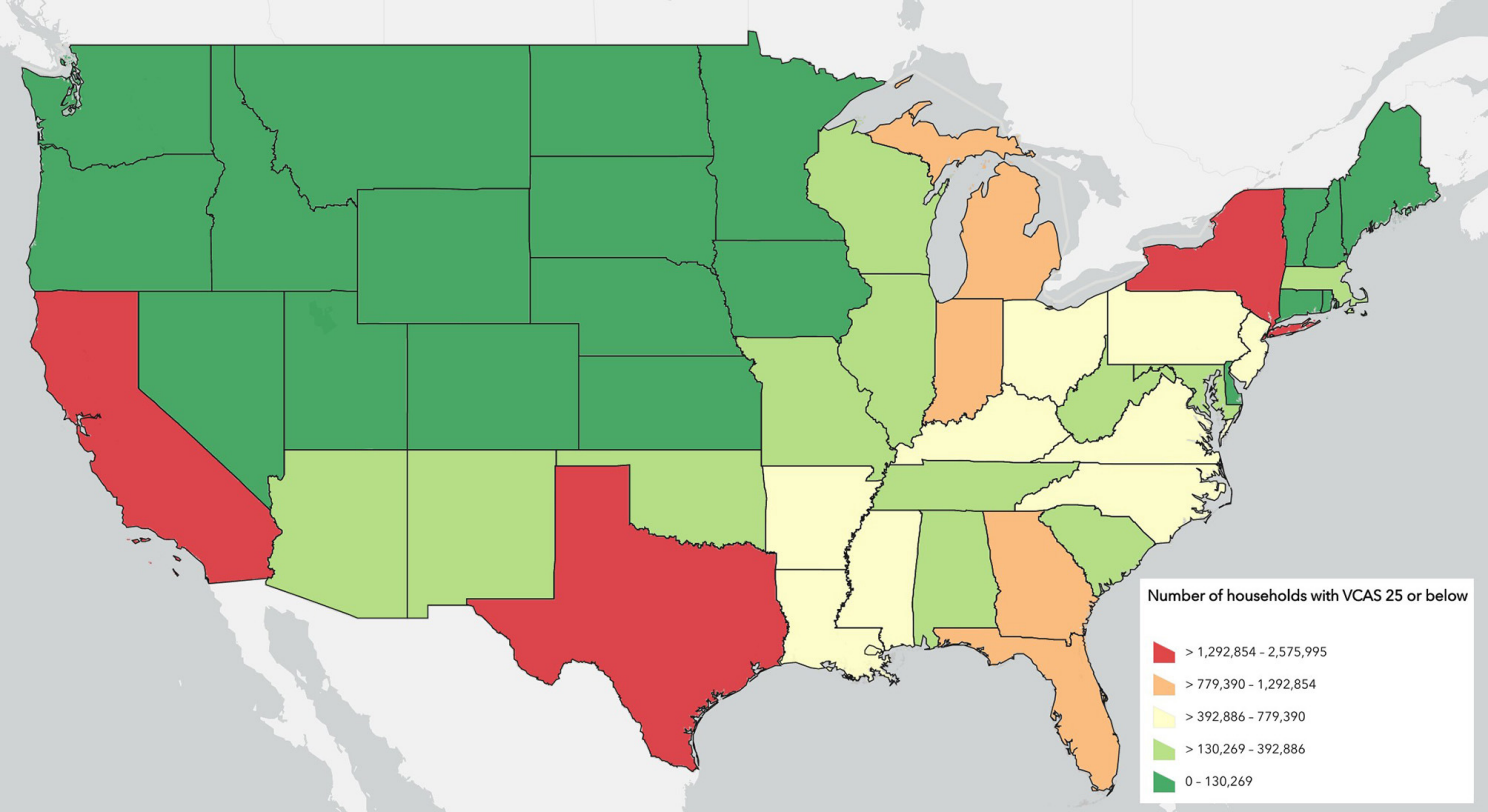
Count of Households in each state that fall within counties in the lowest quartile of the Veterinary Care Accessibility Score.

Lastly, the raw number of households located within each state that have among the lowest quartile of access to veterinary care at the county level is a final way to view the results. Some counties with low access may also have a small population while others may have very high populations. This way of viewing the data can help to quantify the relative order of magnitude of need in any given state relative to other states. Figure 4 summarizes the count of households that are located in counties within the lowest quartile of the VCAS aggregated by state.

## Discussion

There are noticeable regions that have low access to veterinary care as visible on the previous figures. These areas may be optimal opportunities to expand access to care services. The results can also help bring attention to a few different aspects of the challenges surround access to veterinary care. For example, considering the confluence of the factors used to assign scores is indicative of the complexity of the access to chare challenge. It also can be used to show the magnitude of the problem. For example, the results have identified that there are just over 21 million households residing within counties ranked in the lowest (least accessible) quantile, representing an estimated 25.2 million companion animals. As such, the VCAS represents an important snapshot into the challenges of access to veterinary care across the contiguous US. It highlights places of opportunities for programs aimed at increasing access for underserved populations. It also provides a tool for policymakers as they consider how policies could be used to encourage better access to veterinary care.

While the VCAS could be used in a variety of contexts, it lends itself to four potential use cases by different stakeholders in the access to veterinary care arena: (1) Animal welfare funding agencies could use the VCAS to help focus efforts and resources in areas the greatest potential for impact; (2) Service providers (for-profit and nonprofit) could use the VCAS to identify potential markets for new or expanded services; (3) Policymakers could use the VCAS to gain insight into the accessibility of veterinary care in their communities, and address deficiencies through policy changes and other programs; (4) Researchers could use the VCAS to inform further explorations into the topic of veterinary access to care as well as intersections with human healthcare and other “one health” topics. The rankings could also be helpful for animal shelters who are interested in understanding the needs of their communities as it relates to access to care service. Further, shelters may be able to use deficiencies in access to veterinary care in their communities to seek support for programs that increase access as part of initiatives to keep animals in their homes and avoid shelter surrender.

Many questions remain about what an optimal distribution of veterinary clinics would be. This is confounded by the fact that simply having physical access to a clinic does not actuate true access due to the other variables discussed in the index formulation. Different communities would likely need different solutions. The problem of access to care is enormous and the solutions will thus need to take different forms dependent on available resources and the socioeconomic conditions in the individual communities. The VCAS represents a starting point in conceptualizing potential solutions based on the unique traits of individual communities.

There are some distinct limitations to this study. Any choice of a political geographic unit does present a modifiable areal unit problem because the shape and size of the unit of analysis can impact the results of the spatial analysis (32). An ecological fallacy can be also be a concern, particularly when considering access to care in aggregate. For example, one cannot assume that everyone in a county has high access to care, despite the county as a whole having high access. Some counties may have very significant levels of income disparity and clinics are not distributed evenly across the landscape in a county. Pockets of very low access may thus nest within even high access communities and vice versa. So what is true for the county is not necessarily true for every individual household in that county. This research treated all clinics as having equal contribution to the capacity for care in the area of analysis. This is an additional limitation as different types of clinics (specialty, emergency and general care) contribute differently to the access to veterinary care in a community as do hours of operation. State policies regarding the scope of practice of veterinary technicians may also influence how much service is provided per unit of employees. Lastly, mobile clinics may provide service to a broader geographic area.

The likelihood of sharing the home with a companion animal (and the number of animals per home) is not stable over space and can vary by the rural/urban character of a region, family size, income and other factors (29, 33). Thus, the actual number of companion animals in any given county could likely deviate from the estimates used in the index. The explanatory impact of these trends on the density of clinics could be explored in future research.

Lastly, this research presents one picture in time. The socioeconomics, pet populations as well as the locations of clinics and numbers of employees will shift over time necessitating an update of the analysis. Routine updating of the map will be important to maintain its usefulness

There are many opportunities to advance understanding of the geographic distribution of access to care through future research. Advances in understanding the distribution of pets across the landscape would be one important example. Additional understanding of the barriers to access to care could also improve the index particularly as it relates to differing levels of care (preventative, sick, emergency etc) or income and education levels of pet guardians. Examining the geography at other units of analysis, such as census tract, would refine understanding of the distribution and the functional impact of spatial disparities. Understanding the optimal number of employee to pet ratio would not only allow the index to be conceptualized more absolutely, and less relative, it would also provide important parameter specification for other geographic approaches to evaluation access to care, particularly at finer scales. Further research into the distribution of different types of clinics, such as emergency care, would also add to the understanding of this complex issue.

## Data Availability Statement

The data analyzed in this study is subject to the following licenses/restrictions: data from AVMA and ESRI are owned by those entities and so cannot be shared. Requests to access these datasets should be directed to sue@accesstovetcare.org.

## Author Contributions

SN: project conceptualization, data extraction and analysis, and manuscript writing. MG: project conceptualization, edited manuscript, and created the figures. Both authors contributed to the article and approved the submitted version.

## Conflict of Interest

The authors declare that the research was conducted in the absence of any commercial or financial relationships that could be construed as a potential conflict of interest.

## Publisher's Note

All claims expressed in this article are solely those of the authors and do not necessarily represent those of their affiliated organizations, or those of the publisher, the editors and the reviewers. Any product that may be evaluated in this article, or claim that may be made by its manufacturer, is not guaranteed or endorsed by the publisher.
